# Cost and cost effectiveness of long-lasting insecticide-treated bed nets - a model-based analysis

**DOI:** 10.1186/1478-7547-10-5

**Published:** 2012-04-04

**Authors:** Anni-Maria Pulkki-Brännström, Claudia Wolff, Niklas Brännström, Jolene Skordis-Worrall

**Affiliations:** 1 UCL Centre for International Health and Development, UCL Institute of Child Health, 30 Guilford Street, WC1N 1EH London, UK; 2 Department of Economics, Stockholm School of Economics, P.O. Box 6501, Stockholm 118 83, Sweden; 3 Department of Mathematics, University of Helsinki, Helsinki 00014, Finland

**Keywords:** Insecticide-treated bed nets (ITN), Long-lasting insecticide-treated bed nets (LLIN), Replenishment, malaria

## Abstract

**Background:**

The World Health Organization recommends that national malaria programmes universally distribute long-lasting insecticide-treated bed nets (LLINs). LLINs provide effective insecticide protection for at least three years while conventional nets must be retreated every 6-12 months. LLINs may also promise longer physical durability (lifespan), but at a higher unit price. No prospective data currently available is sufficient to calculate the comparative cost effectiveness of different net types. We thus constructed a model to explore the cost effectiveness of LLINs, asking how a longer lifespan affects the relative cost effectiveness of nets, and if, when and why LLINs might be preferred to conventional insecticide-treated nets. An innovation of our model is that we also considered the replenishment need i.e. loss of nets over time.

**Methods:**

We modelled the choice of net over a 10-year period to facilitate the comparison of nets with different lifespan (and/or price) and replenishment need over time. Our base case represents a large-scale programme which achieves high coverage and usage throughout the population by distributing either LLINs or conventional nets through existing health services, and retreats a large proportion of conventional nets regularly at low cost. We identified the determinants of bed net programme cost effectiveness and parameter values for usage rate, delivery and retreatment cost from the literature. One-way sensitivity analysis was conducted to explicitly compare the differential effect of changing parameters such as price, lifespan, usage and replenishment need.

**Results:**

If conventional and long-lasting bed nets have the same physical lifespan (3 years), LLINs are more cost effective unless they are priced at more than USD 1.5 above the price of conventional nets. Because a longer lifespan brings delivery cost savings, each one year increase in lifespan can be accompanied by a USD 1 or more increase in price without the cheaper net (of the same type) becoming more cost effective. Distributing replenishment nets each year in addition to the replacement of all nets every 3-4 years increases the number of under-5 deaths averted by 5-14% at a cost of USD 17-25 per additional person protected per annum or USD 1080-1610 per additional under-5 death averted.

**Conclusions:**

Our results support the World Health Organization recommendation to distribute only LLINs, while giving guidance on the price thresholds above which this recommendation will no longer hold. Programme planners should be willing to pay a premium for nets which have a longer physical lifespan, and if planners are willing to pay USD 1600 per under-5 death averted, investing in replenishment is cost effective.

## Background

Distributing insecticide-treated bed nets (ITNs) has become an integral component of national anti-malaria programmes e.g. [1]. Within those programs, the World Health Organization (WHO) [2] now recommends full coverage of long lasting insecticide-treated nets (LLINs), which means that each household should own one LLIN for every two people living there [3]. LLINs were developed in the 1990s and first approved by the WHO Pesticides Evaluation Scheme (WHOPES) in 2003. With the entry of new manufacturers in recent years, LLINs have become cheaper and more readily available. An LLIN now costs around USD 4.5 [4-6] and provides effective insecticide protection for at least 3 years^a^. By comparison, a conventional net costs considerably less and must be retreated after 2-3 washes, or about every 6-12 months.

Few economic evaluations of LLIN distribution have been published. Mueller et al. [7] found that distributing LLINs within a measles vaccination campaign in Togo compared favourably against other public sector ITN programmes. Kolaczinski et al. [4] studied variation in usage and delivery cost in Uganda. Yukich et al. [1] used data from Togo and four conventional ITN programmes and concluded that in 3 of 5 cases, 5-year LLINs were cost effective compared to conventional ITNs and to 3-year LLINs. Other LLIN programmes have been evaluated for programme effect only [8-12] with the most common measures of effect being household bed net ownership and usage among children under 5.

According to the WHO's World Malaria Report 2010, 254 million ITNs, most of them LLINs, were distributed in sub-Saharan Africa between 2008 and the first three quarters of 2010, with a further 25 million ITNs scheduled for delivery by the end of 2010 [13]. Countries which have launched large-scale LLIN distributions include Togo, Madagascar, Zambia, Uganda, Nigeria, Sierra Leone, Kenya, Niger, Rwanda, Tanzania, Ethiopia, the Democratic Republic of Congo, and the state of Orissa in India. However, other malaria programme managers may be put off by the higher price of LLINs and may be uncertain about their cost effectiveness relative to conventional ITNs.

Given the paucity of prospective data and the need for an economic evaluation of LLINs that may be valid in multiple settings, we constructed a simple model of the cost effectiveness of LLINs. Our results suggest that programme planners should be willing to pay a premium for LLINs over conventional nets, especially when LLINs have a longer lifespan, and even when most conventional nets are regularly retreated and retreatment kits are inexpensive. More generally, we show how LLIN programme cost effectiveness varies with bed net price, lifespan, usage and loss rates. We also estimate that in addition to the replacement of all nets every 3-4 years, distributing a smaller number of replenishment nets each year is incrementally cost effective if planners are willing to pay USD 1600 per under-5 death averted. Our model is intended to represent the costs of a large-scale national programme over a relatively long time horizon (10 years).

## Methods

### Literature review

Pubmed, JSTOR and Web of Science were searched using the key words "malaria nets", "malaria cost and benefit analysis", and "malaria nets cost effectiveness analysis"; and "malaria nets" in combination with "intervention", "cost analysis", "cost-benefit analysis", "cost effectiveness analysis", "cost of illness", "cost savings", "distribution cost", "economic evaluation", "household costs" and "primary prevention". References of all identified articles were then hand-searched to identify additional articles. The main literature search was conducted between May and July 2010 and was restricted to peer-reviewed articles published in 1990 or later. Where the peer-reviewed article referred to a non-peer reviewed article for more detailed results, we also consulted the latter if necessary e.g. [1,14]. No language limiters were used in the search, however articles in a foreign language were not read except for any English-language abstracts.

### Model specification

Determinants of the cost effectiveness of bed net distribution were identified from the literature review described above as: price and lifespan of nets; procurement and delivery costs; insecticide retreatment costs (conventional ITNs only); replenishment need and costs; effectiveness of nets; coverage and usage rates. We derived parameter values for bed net effectiveness, coverage and usage rates, delivery cost, and retreatment need and cost from the literature review. A base case was constructed whereby a large-scale programme achieves high coverage and usage throughout the population by distributing either LLINs or conventional nets through existing health services, and retreating a large proportion of conventional nets regularly at low cost. This base case model served as a comparator against which the effect of alternative parameter values and assumptions was explicitly explored. In particular, the effect of replenishment was explored through three alternative scenarios. The values used in the base case model, one-way sensitivity analyses and replenishment scenarios are summarised in Table 1.

**Table 1:** Base case and sensitivity analysis values and sources

Variable	Base case	Sensitivity analysis	Sources
**Protective effectiveness of ITNs**	5.5 all-cause deaths are prevented per 1000 under-5 s protected every year	- (estimate only relevant if usage is high)	[35]

**Coverage**: number of nets relative to population size	1 million nets distributed to 4 million people, 20% aged under 5	-	-

**Usage rate **in first year after bed net distribution	50% overall 70% under-5 s	30% overall and 50% under-5 s; 30% both overall and under-5 s	See Methods - Programme effect

**ITN purchase price**	USD 4	USD 3 - USD 7 (LLINs) USD 1 - USD 5 (conventional nets)	[4]

**Bed net lifespan**	3 years	3 - 5 years (LLINs) 1-4 years (conventional nets)	[41,43]

**Delivery cost and method**	USD 1.4 per net Large-scale free integrated distribution	USD 3.86 per net Subsidised sales and social marketing	See Table 2

**Insecticide retreatment **(conventional nets only)	75% of nets are treated annually for USD 0.64 per net	50% treated annually or 75% treated biannually for USD 1.28 per net	See Table 3

**Replenishment need**	No replenishment need	Increasing/constant/decreasing proportion of nets is lost each year.	See Methods -Replenishment

**Replenishment net delivery cost**	-	USD 1.4 or USD 3.86 per net	See Table 2

### Programme effect

To enable comparison of programme effect in a number of settings, the main effect measure in our model is the number of people protected. "People protected" refers to the number of people who, on an average night, sleep under an ITN. The strength, and weakness, of this measure is that it does not distinguish between users (adult or child) nor does it require information of the local transmission rate.

Our model assumes that the bed net programme targets a population of 4 million people of whom 20% are aged under 5. Given this arbitrary population, we have calculated programme effect using assumptions about programme coverage, bed net usage and protective effect that are based on studies of previous ITN programmes and current data on coverage and usage rates. The base case parameter values are summarised in Table 1. The calculation of programme effect (number of people protected and under-5 deaths averted) is detailed at the end of this section.

The RBM Partnership and the UN Secretary-General called for universal coverage of malaria prevention and treatment by the end of 2010. This would mean one LLIN for every two people (coverage) and at least 80% of people at risk from malaria using LLINs (usage) [3]. The RBM website provides an updated Progress Report, which at the time of writing suggested that 25 countries had reached 80% usage in their target populations [15]. WHO [13] estimates that 42% of African households owned at least one ITN and usage among under-5 s was 35% in 2010. Certainly the most recent Malaria Indicator Surveys (MIS) [16] indicate that coverage and usage have increased considerably from levels reported in Demographic and Health Surveys (DHS) [17] conducted only few years earlier. However, the three MIS reports from 2010 available at the time of writing (Kenya, Malawi and Zambia) suggested the targets were still some way away: on average, 58% of rural households owned an ITN, and 49% of under-5 s and 45% of pregnant women slept under an ITN.

Our base case assumes that a usage rate of 50% in the general population is achieved by distributing one net for every four people. We assume that 20% of the population are aged under 5, which gives a coverage of 0.8 under-5 s per net. This is comparable to 0.9 reported by Mulligan et al. [18] and 1.0 assumed by Yukich et al. [1]. Small-scale campaigns typically achieve higher levels of coverage than 4 people per net (e.g. 1.3-2.4 people per net in [4,19,20].

Our base case values of 4 people per net and 50% usage are lower than the RBM universal coverage target levels yet not dissimilar to levels suggested by those who argue that relatively high coverage of the whole population is the most effective way to protect vulnerable groups because of community effects [21-23]. Community effects exist if bed nets benefit non-users through a reduction in the local parasite prevalence [24-29]. This requires relatively high coverage and usage rates; e.g. 35-65% usage [23] or that over 50% of households own enough ITNs to cover all household members levels, see also [28,29].

Our assumption of 70% usage rate among under-5 s is also below the RBM goal but optimistic given actual usage rates measured after bed net distributions. Rates above 60% are rarely reported (e.g. 52% [8], 36-81% [10], 56% [11]) although [30-32] report usage rates above 75%. There is some evidence to suggest that usage falls over time [33] however this body of evidence is small; therefore we assume that the proportion of nets used is constant. In sensitivity analysis, we examine 30% overall usage and 50% and 30% under-5 usage.

In the base case model in which there is no replenishment need, LLIN programme effect is constant and calculated as

(1) $$PP_{LLIN}=POP*USE*10$$

*PP_LLIN _*= number of people protected using LLINs over 10 years

*POP *= population

*USE *= average usage rate (%)

Conventional ITN programme effect^b ^over the 10-year period is calculated as:

(2) $$PP_{ITN}=\left[R*NETS+RETREAT\right]*POP*USE/NETS$$

*PP_ITN _*= number of people protected using conventional ITNs over 10 years

*R *= number of ITN distribution rounds in the 10-year period

*NETS *= number of nets in each round

*RETREAT *= number of retreatments delivered between rounds

The first half in square brackets represents "treated net-years" and the second half can be interpreted as the number of people protected per net distributed.

When LLINs are lost over time and not replenished, programme effect is the average number of people protected over a 10-year period. For each year *t*,

(3) PP(t)=LOSS(t)*POP*USE

LOSS(t) is the loss rate, see section Methods - Replenishment. When lost LLINs are annually replenished, we assume that programme effect is the same as if there was no replenishment need (1).

There is general consensus in the literature that ITNs reduce malaria morbidity and mortality. Most evidence concerns all-cause mortality, particularly among children under 5 years old, although estimates of mortality and morbidity directly attributable to malaria are also available [10,34]. The Cochrane review by Lengeler [35] estimates that 5.5 all-cause deaths are prevented each year for every 1000 under-5 children protected. This estimate is based on small-scale studies in malaria-endemic areas with high bed net coverage, and therefore serves as an upper bound with a need for sensitivity analysis on this value. Studies published after the Cochrane review are not directly comparable, e.g. [36] only report malaria cases averted and [37] focus on long-term effects when ITN use was relatively low.

In the base case with high usage among children, we multiply the number of under-5 s protected each year by 5.5 per 1000 [35] to obtain the number of under-5 deaths averted, DA(t):

(4) DA(t)=5.5*UP(t)/1000

*UP(t) *= no. under-5 s protected; calculated as (1) but for the under-5 population using the under-5 usage rate.

We then assign 33 DALYs to each death of a child under-5, giving the number of DALYs averted each year (DALY(t)) as

(5) DALY(t)=33*DA(t)

This is equivalent to treating all child deaths as infant deaths and discounting at 3% per annum [1,38]. Thereby we follow the common approach in the bed net literature that DALYs averted are calculated from deaths only e.g. [1,39].

### Programme cost

Following the literature review, we decided upon a prevention-only model and a modified provider perspective such that the costs of treating malaria were excluded. We assume that each bed net is characterised by its purchase price, physical lifespan and the duration of insecticide protection.

Sensitivity analyses tend to find that bed net price and lifespan have a significant impact on programme cost effectiveness e.g. [4,40]. However, the definition of lifespan is not universally agreed. In particular, should the average lifespan in the field reflect the proportion of nets lost to accidents or used for other purposes? We follow WHO [13] and define LLIN lifespan simply as the period during which LLINs retain full efficacy. The lifespan of a conventional ITN is the period for which, if regularly retreated, the net provides effective protection against malaria. In our model, lifespan determines the frequency of bed net distributions and the period over which bed net costs are annualised.

In the base case model, we assume each bed net - whether conventional or long-lasting - has a 3-year lifespan and costs USD 4. For an LLIN to be approved by the WHO Pesticides Evaluation Scheme (WHOPES), at least 80% of the nets must pass tests for effectiveness after being used by households in randomised field trials lasting at least 3 years [41]. If a manufacturer proposes say a 5-year lifespan, 80% of nets must pass the tests after 5 years [42]. WHO [13] also assumes an average ITN lifespan of 3 years, and a maximum of 5 years. Evidence on physical lifespan other than from WHOPES reports comes mainly from small-scale research trials; e.g. Erlanger et al. [43] found that conventional ITNs subject to daily wear and tear lasted two to three years.

WHO's Global Price Reporting Mechanism [6] provides recent LLIN transaction price data^c^. For the 173 orders placed in 2010, the average unit price was USD 4.75 (range USD 3.3 to USD 7.9). There was no obvious relationship between unit price and order size (average USD 4.72 for orders of more than 100,000 LLINs). In sensitivity analysis, we vary LLIN lifespan between 3 and 5 years and unit price between USD 3 and USD 7. The choice of base case price is further supported by UNICEF [5] who report a weighted average price of about USD 4.5 for the first three quarters of 2010 and Kolaczinski et al. [4] who report recent quotes of USD 3.90 and USD 4.50 obtained by the Malaria Consortium (USD 5.50 for small volume procurement). In sensitivity analysis, we allow conventional nets to have a lower purchase price (USD 1 - USD 5) and/or shorter lifespan (1 - 4 years) than LLINs. The WHO database [6] provides only one record of an order for conventional nets ("non-treated bednet") in 2010 for which the unit price was USD 2.2.

We assume that new nets (of the same type) are distributed when nets reach the end of their lifespan. The same number of nets is distributed in each of these "main" rounds, and the cost of each round is given by NETS * (bed net purchase price + domestic delivery cost per net). In addition, lost nets may be replenished through small annual distributions between main rounds. Conventional ITN programmes also incur the cost of retreating nets. See separate sections on Delivery cost, Replenishment, and Insecticide retreatment. Total programme cost is the sum of main round, replenishment and retreatment costs over 10 years less the value of bed nets with lifespan remaining. Value remaining is a share of the (discounted) last main round cost, with the share equal to the proportion of lifespan remaining, e.g. two-thirds for 3-year nets distributed in year 9.

Bed net price and delivery cost were annualised over bed net lifespan using a discount rate of 3%. All costs were discounted to year zero using a discount rate of 3%. Exchange rates presented in the original sources and the US consumer price index were used to convert all costs extracted from the literature to 2009 US dollars.

### Replenishment

The loss of nets over time reduces programme effect and raises the potential need to distribute replenishment nets. Nets may be lost because they i) are severely damaged through wear and tear, fire or other accident; ii) are used for other purposes; or iii) are sold or given away to relatives. However, we found no published evidence on the cost of replenishment, and very little on replenishment need after the first year of use. Hassan et al. [44] found that 131/142 (93%) of households who had received LLINs still owned them 1.5 years later, although some of these nets were totally damaged. WHO [13] assumes that on average 4% of nets are discarded each year.

We examine three different scenarios of the replenishment need, which may or may not be met by distributing new nets. Scenario 1 is based on a model developed by Albert Kilian^d ^and assumes that the rate at which nets are lost has an S-shape: it increases over time, until 50% of the originally distributed nets are remaining, and subsequently slows down. We applied the model assuming that 50% of nets have been lost when the nets come to the end of their lifespan. The proportion of nets remaining in preceding years is given by:

(6) $$\frac{\text{no}\text{. nets remaining (t)}}{\text{no}\text{. nets distributed (t = 0)}}=\exp\left(k-\frac{k}{1-{\left(t/L\right)}^{2}}\right)$$

where *k *is the bed net lifespan (in years) and *t *is the number of years since the distribution. *L *is a parameter that depends on lifespan. First we solve for *L *for values of *k *between 2 and 5 using the assumption that when *t = k*, 50% of nets are remaining. Given the value of *L*, the proportion of nets remaining at any time *t *is then easily computed. For example, for nets with a 3-year lifespan, 93.8% are remaining in the second year after the distribution, 76% in the third year, and 50% in the fourth year.

In Scenario 2, the annual loss is a constant proportion of the nets originally distributed i.e. each net has a constant probability of being lost. We assume that the same number of nets are lost each year, equal to 5% or 10% of the nets originally distributed. Scenario 3 is similar to the one assumed in WHO [13]: a constant proportion - we assume 5% or 10% - of nets currently owned is lost each year and thus the probability of being lost decreases over time.

We make several simplifying assumptions regarding the structure of a replenishment programme. First, the replenishment need is met through annual replenishment distributions. Second, replenishment nets are bought at the same time as nets used in the main distribution round, and are stored at negligible cost until distributed. Third, the replenishment delivery cost per net is the same as in the main round (Table 2), although distribution method may differ. Finally, all nets including replenishment nets are replaced at each main distribution round. We consider the simplicity of these assumptions justified as a first attempt to include a replenishment programme into a model of ITN cost effectiveness.

**Table 2:** Delivery cost estimates

Delivery method	Delivery cost per net (average and range; USD 2009)	Country and source
**Free campaign distribution (collection points or door-to-door)**	2.73 (0.7, 9.0)	Uganda* (separate data for two districts) [4], Kenya [19], Ghana [45], India [36]

**Free distribution integrated to routine services (small scale)**	2.65 (1.66, 3.95)	Burkina Faso [40], Uganda* [4], Dem. Rep. Congo [51]

**Free distribution integrated to routine services (large scale)**	1.40 (0.78, 1.81)	Malawi† and Togo [14]; Eritrea [46]

**Subsidised Sales and Social Marketing**	3.86 (1.34, 7.87)	Tanzania†† [14,39,47], Malawi† [50], Burkina Faso [40], Senegal [14]

Notes to Table 2. Studies that reported financial costs only e.g. [22,62] were excluded.

* For Uganda, [4] report costs for two delivery channels in two districts: campaign delivery in both districts, and delivery through antenatal care in one district.

† The Malawi programme relied more heavily on sales in the early years and routine service delivery later on. We classify Stevens et al. [50] as sales but Yukich et al. [14] as integrated delivery because the latter include two additional years of data.

†† For Tanzania we use different sources for different programmes: Yukich et al. [14] for SMARTNET, Mulligan et al. [47] for TNVS, and Hanson et al. [39] for KINET.

### Delivery cost

The evidence on distribution cost is relatively scarce, although recent evidence has emerged suggesting it varies with the method of distribution. Kolaczinski et al. [4] found that the antenatal care channel (USD 4.5 per net delivered) was more expensive than two targeted distribution campaigns (USD 3.7 and USD 3.0 respectively, 2009 USD). De Allegri et al. [40] estimated that distribution cost in Burkina Faso was USD 4.8 per net (2006 USD) for both the antenatal care channel and subsidised sales.

We identified 17 studies that specified the distribution method and reported economic cost data in sufficient detail for us to calculate "delivery cost" per net, defined in [1,4,14] as: *Delivery cost per net = (Programme economic cost - Price of nets - Price of insecticide)/Number of nets delivered*.^e ^We divided our delivery cost estimates into three groups depending on the distribution method used in the original study, and calculated the average cost of each method. The three different methods represented in the 17 studies are broadly described as: free campaign distribution (nets are supplied at village collection points or delivered directly to the doorstep), free distribution integrated into routine services (typically antenatal care), and subsidised sales supported by social marketing. The average costs and sources are reported in Table 2.

Our data suggests scale effects: the seven larger programmes (750,000 nets or more) cost on average USD 2.2 per net and at most USD 4.7 [18], while the maximum for smaller programmes (at most 65,000 nets) was USD 9.0 [19] and the average USD 3.3.

Our base case delivery cost is USD 1.4 per net, the average for large-scale integrated distribution. Although small compared to De Allegri et al. [40] and Kolaczinski et al. [4], our figure is in fact likely to be an overestimate because only the cost of insecticide, not the full cost of delivering retreatments, is subtracted. Two small-scale studies [19,45] separately reported insecticide and total retreatment costs. The proportion of insecticide commodity cost in total retreatment cost was 27% and 79% respectively (Table 3), which gives little indication as to the likely size of the bias. Overestimation of delivery cost means that nets which are more frequently distributed (i.e. have a shorter lifespan) will appear somewhat more expensive. However, the size of this bias is likely to be small given that the proportion of delivery cost in total cost is small. The sensitivity of our results to delivery cost is examined in sensitivity analysis.

**Table 3:** Insecticide retreatment cost estimates

Scale	Retreatment frequency	Cost of insecticide per retreatment kit (USD 2009)	Total cost per retreatment (USD 2009)	Country and source
**Small scale (≤ 65,000 nets)**	Biannual	0.21	0.78	Kenya [19]
	
	Biannual	0.69	0.87	Ghana [45]
	
	Continuous sales	1.67	-	Tanzania† [39]
	
	Initial impregnation only	0.32	-	India [36]

**Large scale (≥ 0.75 m nets)**	Annual	0.67	-	Malawi†† [14]
	
	Annual	0.23	-	Eritrea [46]
	
	Continuous sales	2.05	-	Senegal [14]
	
	Continuous sales	0.61	-	Tanzania† [14]
	
	Biannual	0.80 (1.16 for kits actually used)	-	Tanzania† [47]

Notes to Table 3. † For Tanzania we use different sources for different programmes: Yukich et al. [14] for SMARTNET, Mulligan et al. [47] for TNVS, and Hanson et al. [39] for KINET.

†† We use Yukich et al. [14] rather than Stevens et al. [50] because the former include two additional years of data.

Table 2 suggests that subsidised sales (with social marketing) and campaign delivery are more expensive on average than routine service delivery. We use USD 3.86 (subsidised sales) in the sensitivity analysis, because all the five examples of free campaign delivery are small-scale campaigns.

### Insecticide retreatment costs and frequency

In order not to bias our findings against conventional ITNs, the base case in our model represents a "best case scenario" with respect to the retreatment of conventional ITNs. We assume that 75% of conventional nets are successfully retreated annually at a cost of USD 0.64 per net treated.

Our literature search revealed two small-scale studies which report a cost of USD 0.8 - 0.9 per retreatment [19,45]. Five other studies report the commodity cost of insecticide [14,36,39,46,47]. The difficulties in separating out the full costs of retreating conventional ITNs from other programme costs are discussed in Yukich et al. [1,14]. To make use of the available data, we estimated retreatment cost from the seven studies by dividing insecticide cost by the number of retreatments and initial impregnations. The median cost is our base case estimate. The sources and estimates are reported in Table 3.

Only considering the commodity cost of insecticide means that we underestimate the true cost of conventional ITNs. In the sensitivity analysis, we therefore double the cost to USD 1.28, which reflects the Yukich et al. [14] estimate that the share of insecticide in total retreatment cost is 50%; Wiseman [19] and Binka [45] report 27% and 79% respectively.

The paucity of evidence on retreatment cost may partly be explained by low retreatment rates. Mulligan et al. [18] found that 69% of mothers given a retreatment kit used it to retreat their nets. Marchant et al. [48] found that low retreatment was the main reason for low ITN coverage. Armstrong Schellenberg et al. [49] conclude that most of those households who had tried retreatment did not make a regular habit of it. Indeed, the WHO (2008) cited difficulties in effectively delivering retreatment as a reason for recommending the move conventional nets to LLINs. In sensitivity analysis, we reduce the retreatment rate from 75% to 50%.

Typically the initial impregnation and subsequent retreatments are assumed to provide either 6 months or 1 year of protection e.g. [1,47,50] and the other value is assumed in sensitivity analysis. This assumption can have a significant effect on programme cost effectiveness. For example, Yukich et al. [1,14] conclude that if insecticide lasts for one year, conventional nets are as cost effective as LLINs in two out of five large-scale programmes, but if retreatment is required every six months, LLINs are more cost effective in all five cases. Reducing the duration of insecticide protection from 1 year to 6 months has approximately the same effect as doubling the cost of retreatment, which is why we do not separately report on sensitivity analysis for the duration of insecticide protection.

## Results and discussion

### Long-lasting versus conventional ITNs in the base case

LLINs are more cost effective than conventional ITNs in the base case in which there is no difference in purchase price or lifespan. Each delivered LLIN costs USD 5.14 on average over the 10-year period. A conventional net that is regularly retreated with insecticide costs USD 6.94 on average. The difference of USD 1.80 per net is explained by a higher total cost (due to the additional cost of retreatment) and a smaller programme effect (fewer treated net-years due to a 75% retreatment rate) in the conventional net programme. The annual cost is USD 1.71 per LLIN and USD 2.31 per treated conventional net.

The base case represents a highly effective programme. On average over 10 years, 850,000 of the 1 million conventional nets distributed provide effective protection for 1.70 million people including 476,000 children under 5 each year. The annual cost is USD 1.16 per person or USD 4.13 per child. An estimated 26,180 deaths, equivalent to about 864,000 DALYs, are averted over 10 years.

Using LLINs improves cost effectiveness by 26%. The number of people protected is 2 million each year, including 560,000 children under 5, at an annual cost of USD 0.86 per person or USD 3.06 per child. An estimated 30,800 child deaths, equivalent to some 1.02 million DALYs, are averted over 10 years, at a cost of USD 556 per death or USD 16.8 per DALY averted. The cost saving compared to conventional ITNs is equal to USD 1.07 per under-5 (USD 0.30 per person) protected per year, equivalent to USD 195 per death averted or USD 5.90 per DALY averted. How changes to the parameters of the base case change the above results is discussed in the following sections.

### Bed net price and lifespan

The LLIN is the more cost effective option if LLINs and conventional nets have the same lifespan, unless the LLIN price is above a certain threshold. In the base case, the price differential must be greater than USD 1.5. For example, if the 3-year conventional ITN is priced at USD 2.4 and the 3-year LLIN at USD 4, or the conventional net is USD 4 and the LLIN USD 5.9, then the two nets are equally cost effective. The price differential necessary to change cost effectiveness in favour of conventional nets is larger if the retreatment rate is less than 75%, the retreatment need more frequent, or the retreatment cost larger. For example, if the retreatment rate is 50% (or either retreatment is biannual or the cost per retreatment is USD 1.28), the conventional net must be priced at USD 1.9 (1.6) to be cost effective against the LLIN which costs USD 4.

A longer lifespan also brings delivery cost savings. For example, using 5-year LLINs instead of 3-year LLINs reduces the cost per under-5 death averted by USD 204 in the base case if there is no difference in price. Generally, we find that price can increase by USD 1 or more for each one-year increase in lifespan, if nets are of the same type.

**Figure 1:**
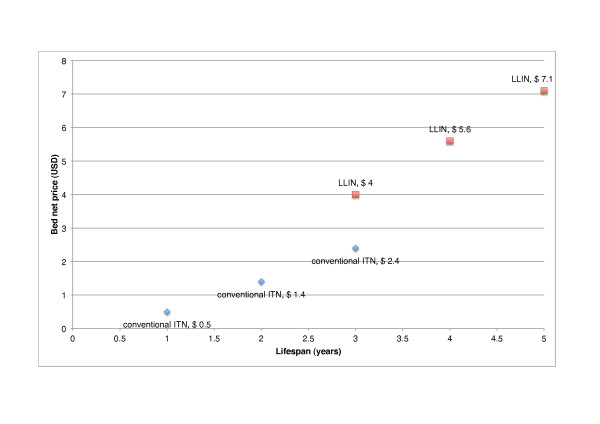
**Equally cost effective bed nets of different price, lifespan and type**. Each bed net choice is as cost effective as the base case, the 3-year LLIN priced at USD 4. Programme cost is USD 1.71 per net delivered (and retreated) per annum, USD 0.85-0.86 per person (USD 3.05-3.06 per under-5) protected per annum, USD 554-556 per under-5 death averted and USD 16.8-16.9 per DALY averted.

Figure 1 illustrates bed nets of different price, lifespan and type which are equally cost effective as the 3-year LLIN at USD 4. In this case, a 4-year LLIN at more than double the price of a 3-year conventional net (USD 5.6 vs. USD 2.4) is equally cost effective, and a 2-year advantage in lifespan can be accompanied by nearly a tripling of the price (USD 7.0). Thus if LLINs can offer a longer lifespan, programme planners should be willing to pay a considerably higher price for LLINs than for conventional nets. As we have demonstrated, this is the case even in programmes which inexpensively retreat a large proportion of conventional nets. The advantage that LLINs have over conventional nets - that they do not have to be retreated - is magnified for each additional year of useful life.

### Importance of usage rate for programme effect

**Figure 2:**
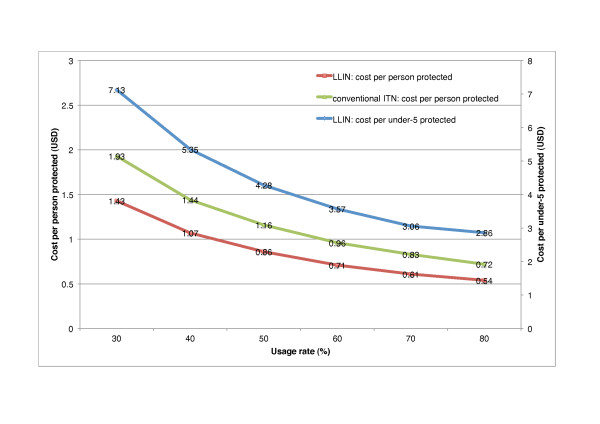
**Relationship between usage rate and programme cost effectiveness when programme cost is constant**. The figure illustrates how cost effectiveness changes depending on how frequently nets are used. The base case is 50% usage among the general population and 70% among under 5s, which gives LLIN cost effectiveness as USD 0.86 per person protected and USD 3.06 per under-5 protected. If conventional nets are used instead (with the same physical lifespan and purchase price and base case assumptions about retreatment), cost effectiveness is USD 1.16 per person protected. A higher usage rate implies a lower cost per person protected.

Programme effect falls proportionally with reductions in the usage rate. Given the same number of nets as in the base case, a 40% reduction in the usage rate from 50% to 30% means 0.8 million (40%) fewer people are protected each year compared to the base case. Consequently, cost per person protected rises to USD 1.43 per year from USD 0.86 in the base case (63% increase). This relationship between the usage rate and programme cost effectiveness is illustrated in Figure 2. If the programme uses conventional nets, the same reduction in usage means that cost per person increases from USD 1.16 to USD 1.93 per year (66% increase) as 680,000 fewer people on average are protected each year.

Our base case with 1 net per 4 people and 50% usage represents a more cost effective programme than one that reaches GMAP and RBM's target coverage and usage values of 1 net per 2 people and 80% usage. This is because with 1 net per 4 people, 40% usage would be sufficient for a programme to be equally cost effective as one with 80% usage and 1 net per 2 people. The comparison is somewhat theoretical however, as in practice distributing a given number of nets to twice the population would be expected to affect programme cost.

Returning to LLINs, if the under-5 usage rate is 50% (30%), 160,000 (320,000) fewer children are protected each year than in the base case (70% usage) and cost per under-5 increases from USD 3.1 to USD 4.3 (7.1) per year. We do not report cost per death averted here because the Lengeler [35] estimate of protective efficacy applies only in high usage settings (see Methods - Programme effect).

### Replenishment

The cost of replenishment depends on the loss rate, the type of nets distributed, and delivery cost. In the following discussion we only consider LLINs in order to abstract from the cost of retreating conventional nets in combination with replenishing lost nets. In Scenario 1, where the number of nets lost increases over time, some 62,000 nets must be distributed in the beginning of the second and 177,000 nets in the third year after each main distribution round in order to keep the number of people protected constant until the next main distribution round in year 4. Assuming nets are priced at USD 4 and the delivery method is the same as in the main rounds (delivery cost of USD 1.4 per net), the six replenishment rounds over 10 years increase overall programme cost by 26%. In Scenarios 2 and 3 a constant proportion of nets distributed/remaining is replenished annually. If this is 10%, replenishment increases programme cost by 22%. Halving the annual replenishment need to 5% also halves the increase in cost to 11%.

Scenario 1 indicates a larger replenishment need than Scenarios 2 and 3 if lifespan is less than 4 years. If lifespan increases, the three scenarios converge: fewer nets are needed in Scenario 1 while more are needed in Scenarios 2 and 3. 4-year nets require seven replenishment rounds, which implies one additional replenishment round in Scenarios 2 and 3 but 72,000 fewer nets overall in Scenario 1 (37,00 nets in year 2, 106,000 in year 3 and 162,000 in the year before the next main distribution). In this case, the S-shape assumption and a 10% annual replenishment need both imply that replenishment increases total cost by 33%.

In Table 4 we present the incremental cost effectiveness of replenishment in three scenarios and for both 3- and 4-year LLINs. Scenario 2 has the smallest annual replenishment need: 5% of distributed nets. Replenishment increases programme cost by 11-26% (17-33%) if lifespan is 3 (4) years. Note that the cost of replenishment in Scenario 2 is now exactly half of that in Scenario 3. Replenishment increases the number of under-5 deaths averted by 5-10% (7-14%) if lifespan is 3 (4) years. A programme without replenishment is somewhat more cost effective; for example, USD 701 vs. USD 611 per death averted in Scenario 1. The cost of replenishment is USD 17-25 per additional person protected per annum or USD 1080-1610 per additional under-5 death averted.

**Table 4:** Impact of replenishment on LLIN programme cost and cost effectiveness

Result	Lifespan	Scenario 1: S-shaped loss rate	**Scenario 2: 5% of distributed nets lost p.a**.	**Scenario 3: 10% of remaining nets lost p.a**.
**Number of replenishment nets required**	3 years	717,000	300,00	600,000
	
	4 years	644,600	350,000	700,000

**Total replenishment cost (delivery cost)**	3 years	USD 4.47 m (USD 906,000)	USD 1.87 m (USD 382,000)	USD 3.75 m (USD 767,000)
	
	4 years	USD 4.32 m (USD 816,000)	USD 2.17 m (USD 443,000)	USD 4.34 m (USD 886,000)

**Impact of replenishment on programme cost**	3 years	+26%	+11%	+22%
	
	4 years	+33%	+17%	+33%

**Impact of replenishment** **on programme effect** **(under-5 deaths averted)**	3 years	2780 more deaths averted (+9.9%)	1386 more deaths averted (+4.7%)	2680 more deaths averted (+9.5%)
	
	4 years	3087 more deaths averted (+11%)	2002 more deaths averted (+7.0%)	3764 more deaths averted (+14%)

**Cost per under-5 death averted with (without) replenishment**	3 years	USD 701 (611)	USD 617 (582)	USD 678 (609)
	
	4 years	USD 567 (474)	USD 497 (456)	USD 568 (486)

**Incremental cost effectiveness of replenishment: cost per additional under-5 death averted (per person protected p.a)**	3 years	USD 1609 (25)	USD 1353 (21)	USD 1399 (22)
	
	4 years	USD 1400 (22)	USD 1085 (17)	USD 1154 (18)

Notes to Table 4: All nets are LLINs priced at USD 4 each. Other variables take values specified in the base case model (Table 1).

The incremental cost effectiveness of replenishment is not very sensitive to replenishment delivery cost because this is a relatively small proportion of total replenishment cost. For example, increasing delivery cost by some 2.5 times to USD 3.86 per net (the average for subsidised sales and social marketing, see Table 2) increases the incremental cost of replenishing 3-year LLINs by 34%. The total cost of replenishment increases by 7.0%/3.3%/6.1% in Scenario 1/2/3, giving a cost of USD 33/28/29 per additional person protected per annum or USD 2151/1811/1873 per additional under-5 death averted.

### Limitations

The generalisability of cost effectiveness results from past to future programmes, or across programmes of different scale, is hindered by the relative lack of evidence on some key determinants of programme cost and effect, as well as the complexity of the relationships involved. Our literature review identified the following elements of the full (provider) costs on which there is little or no published evidence: international transport costs and transaction costs relating to the bed net procurement process. We suspect two reasons for why these cost categories have not been discussed: i) the short time scale of most evaluations, and ii) modified provider perspective are the norm and as such, costs incurred by the procuring body (e.g. donor) are not included.

International transport costs do not affect the choice of net as long as manufacturers are located in the same region, however import taxes and tariffs will be relevant for individual countries and for the comparison of programme costs across countries. With the exception of Becker-Dreps et al. [51] and Yukich et al. [1,46], international shipping costs are not discussed in the bed net literature. Yukich et al. [46] use the commodity, insurance and freight (CIF) price of bed nets, and Yukich et al. [1] use either the CIF price or the full retail price plus subsidies. WHO-CHOICE [52] (see also [53]) suggest estimating international shipping costs by multiplying commodity prices by a so-called CIF/FOB mark-up, equal to 25% and 44% for the WHO regions South East Asia D and Africa D respectively. Because the effect of such a mark-up on our results is similar to changes in bed net price, which we already discuss at length, we do not report on the results here. We presume that the lack of data is either because the cost is already included in the price paid for nets or international transport cost is incurred by donors of nets (e.g. Becker-Dreps et al. [51] report that nets donated by the Global Fund were valued at USD 8 including shipping and customs).

Procurement overheads, on which we could not find any evidence, are nevertheless likely to be of increasing relevance to programme planners and evaluations with repeated bed net purchasing and distribution. We would expect that the procurement of bed nets incurs overheads such as the costs of a tender process. We considered the effect of an arbitrary fixed cost of USD 200,000 for every main distribution round on the results in Figure 1 and found that the price of the 5-year LLINs could increase to USD 7.2 but otherwise a cost of this magnitude (3.7% of total programme cost over 10 years) has little impact on the relative cost effectiveness of nets with different lifespans (results not reported). The lack of literature on overheads may be because these costs are absorbed into overall programme cost e.g. [1,19] or again because these costs are commonly incurred by donors. In the long run however, such costs should not be ignored, particularly if local ownership of programmes is being encouraged alongside the sustainability of interventions and if the frequency of required bed net distributions is high.

We treat the usage rate rather simplistically in our model because evidence on its determinants and variation over time is relatively scattered. Consistent with the findings of Yukich et al. [1] and Kolaczinski et al. [4], we assume that the distribution channel does not lead to significant differences in usage or retention. Specifically, paying for a net does not appear to be associated with a greater likelihood of use [54,55].

A limitation of our model is that we do not address issues of equity. Our sensitivity analysis is also limited by the lack of evidence on the efficacy of nets when coverage or usage is low. We have overcome the issue in part by referring to the number of people protected. Other issues around programme effect discussed in the literature but excluded from our model because of the relatively small evidence base are the protective efficacy of untreated nets and nets used beyond their lifespan, and the long-term effects of ITN use. There is some evidence that unprotected nets i.e. conventional ITNs which have not been treated with insecticide in the last 6-12 months, provide protection against malaria [56,57]. Long-term effects are a concern if, without repeated exposure to malaria, malaria infections are simply postponed to later in childhood when the morbidity effects may be even more serious [58,59]. Askjaer et al. [60] find evidence of lower levels and less diversity of antibodies, while others find no evidence of negative long-term effects [26,61].

While our assumption that nets are discarded and replaced at the end of their lifespan may underestimate true programme effect, long-term effects would imply that our model overestimates effect. We thus implicitly assume that the two effects cancel out. We also consider our simplified approach to programme effect justified because of its transparency. As a first step, we would suggest extending our model so that protection offered by replenishment nets is calculated from the date of delivery rather than the date of procurement. However, we expect that our current specification better mirrors true programme cost because programme managers may prefer to use a simple, transparent formula, as we have done here.

## Conclusions

This paper uses a modelling approach to integrate the available evidence on the cost effectiveness of conventional and long-lasting ITNs. Our discussion focuses particularly on those factors which decision-makers facing a choice of nets should consider. Our results support the WHO [2] recommendation to distribute only LLINs, in particular if LLINs have a longer lifespan. Our analysis also identifies the pricing thresholds above which this recommendation will no longer hold.

In our base case, LLINs are 26% more cost effective than conventional nets. The difference is large, considering that we have assumed a successful conventional ITN programme in which a high proportion of nets are retreated at a low cost. The cost saving offered by LLINs is equal to USD 0.30 per person protected per year, or USD 195 per under-5 death averted. If conventional nets and LLINs have the same lifespan (3 years), LLINs are more cost effective unless they are priced at more than USD 1.5 above the price of conventional nets. Because a longer lifespan brings delivery cost savings, each one year increase in lifespan can be accompanied by a USD 1 or more increase in price without the cheaper net (of the same type) becoming more cost effective.

In line with previous studies, we find that cost effectiveness is highly sensitive to the usage rate. If the (LLIN) programme achieves a 50% (30%) under-5 usage rate rather than the 70% assumed in the base case, the cost per under-5 protected is USD 4.3 (7.1) rather than USD 3.1 per year.

We estimate that if programme planners are willing to pay USD 1600 per under-5 death averted, investing in replenishment is cost effective. As LLINs become increasingly common, we suspect that attention of programme planners and academics alike will turn increasingly to the issue of replenishment: what is the replenishment need (or loss rate), how should replenishment be carried out, if at all, and at what cost. Thus more publicly available data on the replenishment need and its determinants would be highly valuable. One concern with the move to LLINs is whether less frequent distribution and the cessation of retreatment activities - as would be the case with LLINs - would negatively affect usage rates. The evidence is currently unable to predict whether this would be the case.

## Abbreviations

CIF: Cost, insurance and freight; DALY: Disability-adjusted life-year; FOB: Free on board; ITN: Insecticide-treated bed net; LLIN: Long-lasting insecticide-treated bed net; RBM: Roll Back Malaria Partnership; WHO: World Health Organization; WHOPES: World Health Organization Pesticides Evaluation Scheme.

## Competing interests

APB, CW and JSW received funding from Bayer S.A.S. (Lyon) to carry out the literature review and construct the model used in this paper. The funder had no role in the literature review, choice of model parameters, or writing of the manuscript.

## Authors' contributions

APB constructed the model, drafted the manuscript and contributed to the design of the study and the literature review. CW carried out the literature review, contributed to drafting the manuscript and revised the model and the manuscript. NB helped to construct the model and critically revised the manuscript. JSW designed and coordinated the study and critically revised the manuscript. All authors read and approved the final manuscript.

## Endnotes

^a^See pages 10-11 for WHOPES approval conditions.

^b^The formula assumes that insecticide protection lasts for 1 year. If duration is 6 months, a multiplier of 0.5 should be used.

^c^We thank a reviewer for suggesting this resource.

^d^Our operationalisation of Scenario 1 directly draws on a formula developed by Nakul Chitnis of the Swiss Tropical Institute. Albert Kilian of the Malaria Consortium shared this formula with us through personal correspondence. Kilian has synthesised the available (but as yet unpublished) evidence on the loss of nets over time.

^e^For studies that only reported annual economic cost, we estimated delivery cost as: (Annual economic cost - Price of nets - Price of insecticide) * Length of programme (years)/Number of nets delivered.
